# Supplementary material for: Resolving the full spectrum of human genome variation using Linked-Reads
Source: Genome Res. 2019 Apr;29(4):635–45. doi: 10.1101/gr.234443.118 (PMC6442396; doi:10.1101/gr.234443.118)
Supplement: Supplemental Material [file supp_gr.234443.118_Supplemental_longranger-2.2.zip › longranger-2.2.tar.bz2!/lr2.2-public/lib/stan/cmdstan/stan/doc/api/html/initialize__state_8hpp_source.html]

Stan: src/stan/common/initialize\_state.hpp Source File


|  |  |
| --- | --- |
|  | Stan  2.5.0  probability, sampling & optimization |


- Main Page
- Related Pages
- Namespaces
- Classes
- Files

- File List
- File Members

All Classes Namespaces Files Functions Variables Typedefs Enumerations Enumerator Friends Macros Pages

- src
- stan
- common

initialize\_state.hpp

Go to the documentation of this file.

1 #ifndef STAN\_\_COMMON\_\_INITIALIZE\_STATE\_HPP

2 #define STAN\_\_COMMON\_\_INITIALIZE\_STATE\_HPP

3

4 #include <string>

5 #include <iostream>

6 #include <math.h>

7

8 #include <stan/math/matrix/Eigen.hpp>

9

10 #include <boost/lexical\_cast.hpp>

11 #include <boost/random/additive\_combine.hpp> // L'Ecuyer RNG

12 #include <boost/random/uniform\_real\_distribution.hpp>

13 #include <boost/random/variate\_generator.hpp>

14

15 #include <stan/model/util.hpp>

16 #include <stan/gm/error\_codes.hpp>

17 #include <stan/common/context\_factory.hpp>

18 #include <stan/common/write\_error\_msg.hpp>

19

20 namespace stan {

21  namespace common {

22

32  template <class Model>

33  bool initialize\_state\_zero(Eigen::VectorXd& cont\_params,

34  Model& model,

35  std::ostream\* output) {

36  cont\_params.setZero();

37

38  double init\_log\_prob;

39  Eigen::VectorXd init\_grad = Eigen::VectorXd::Zero(model.num\_params\_r());

40

41  try {

42  stan::model::gradient(model, cont\_params, init\_log\_prob, init\_grad, output);

43  } catch (const std::exception& e) {

44  if (output)

45  \*output << "Rejecting initialization at zero because of gradient failure."

46  << std::endl << e.what() << std::endl;

47  return false;

48  }

49

50  if (!boost::math::isfinite(init\_log\_prob)) {

51  if (output)

52  \*output << "Rejecting initialization at zero because of vanishing density."

53  << std::endl;

54  return false;

55  }

56

57  for (int i = 0; i < init\_grad.size(); ++i) {

58  if (!boost::math::isfinite(init\_grad[i])) {

59  if (output)

60  \*output << "Rejecting initialization at zero because of divergent gradient."

61  << std::endl;

62  return false;

63  }

64  }

65  return true;

66  }

67

68

81  template <class Model, class RNG>

82  bool initialize\_state\_random(const double R,

83  Eigen::VectorXd& cont\_params,

84  Model& model,

85  RNG& base\_rng,

86  std::ostream\* output) {

87  int num\_init\_tries = -1;

88

89  boost::random::uniform\_real\_distribution<double>

90  init\_range\_distribution(-R, R);

91

92  boost::variate\_generator<RNG&, boost::random::uniform\_real\_distribution<double> >

93  init\_rng(base\_rng, init\_range\_distribution);

94

95  cont\_params.setZero();

96

97  // Random initializations until log\_prob is finite

98  Eigen::VectorXd init\_grad = Eigen::VectorXd::Zero(model.num\_params\_r());

99  static int MAX\_INIT\_TRIES = 100;

100

101  for (num\_init\_tries = 1; num\_init\_tries <= MAX\_INIT\_TRIES; ++num\_init\_tries) {

102  for (int i = 0; i < cont\_params.size(); ++i)

103  cont\_params(i) = init\_rng();

104

105  double init\_log\_prob;

106  try {

107  stan::model::gradient(model, cont\_params, init\_log\_prob, init\_grad, &std::cout);

108  } catch (const std::exception& e) {

109  write\_error\_msg(output, e);

110  if (output)

111  \*output << "Rejecting proposed initial value with zero density." << std::endl;

112  init\_log\_prob = -std::numeric\_limits<double>::infinity();

113  }

114  if (!boost::math::isfinite(init\_log\_prob))

115  continue;

116  for (int i = 0; i < init\_grad.size(); ++i)

117  if (!boost::math::isfinite(init\_grad(i)))

118  continue;

119  break;

120

121  }

122

123  if (num\_init\_tries > MAX\_INIT\_TRIES) {

124  if (output)

125  \*output << std::endl << std::endl

126  << "Initialization between (" << -R << ", " << R << ") failed after "

127  << MAX\_INIT\_TRIES << " attempts. " << std::endl

128  << " Try specifying initial values,"

129  << " reducing ranges of constrained values,"

130  << " or reparameterizing the model."

131  << std::endl;

132  return false;

133  }

134  return true;

135  }

136

137

153  template <class ContextFactory, class Model, class RNG>

154  bool initialize\_state\_source(const std::string source,

155  Eigen::VectorXd& cont\_params,

156  Model& model,

157  RNG& base\_rng,

158  std::ostream\* output,

159  ContextFactory& context\_factory) {

160  try {

161  typename ContextFactory::var\_context\_t context = context\_factory(source);

162  model.transform\_inits(context, cont\_params);

163  } catch(const std::exception& e) {

164  if (output)

165  \*output << "Initialization from source failed."

166  << std::endl << e.what() << std::endl;

167  return false;

168  }

169

170  double init\_log\_prob;

171  Eigen::VectorXd init\_grad = Eigen::VectorXd::Zero(model.num\_params\_r());

172

173  try {

174  stan::model::gradient(model, cont\_params, init\_log\_prob, init\_grad, &std::cout);

175  } catch (const std::exception& e) {

176  if (output)

177  \*output << "Rejecting user-specified initialization because of gradient failure."

178  << std::endl << e.what() << std::endl;

179  return false;

180  }

181

182  if (!boost::math::isfinite(init\_log\_prob)) {

183  if (output)

184  \*output << "Rejecting user-specified initialization because of vanishing density."

185  << std::endl;

186  return false;

187  }

188

189  for (int i = 0; i < init\_grad.size(); ++i) {

190  if (!boost::math::isfinite(init\_grad[i])) {

191  if (output)

192  \*output << "Rejecting user-specified initialization because of divergent gradient."

193  << std::endl;

194  return false;

195  }

196  }

197  return true;

198  }

199

208  bool get\_double\_from\_string(const std::string& s, double& val) {

209  try {

210  val = boost::lexical\_cast<double>(s);

211  } catch (const boost::bad\_lexical\_cast& e) {

212  val = std::numeric\_limits<double>::quiet\_NaN();

213  return false;

214  }

215  return true;

216  }

217

233  template <class ContextFactory, class Model, class RNG>

234  bool initialize\_state(const std::string init,

235  Eigen::VectorXd& cont\_params,

236  Model& model,

237  RNG& base\_rng,

238  std::ostream\* output,

239  ContextFactory& context\_factory) {

240  double R;

241  if (get\_double\_from\_string(init, R)) {

242  if (R == 0) {

243  return initialize\_state\_zero(cont\_params, model, output);

244  } else {

245  return initialize\_state\_random(R, cont\_params, model,

246  base\_rng, output);

247  }

248  } else {

249  return initialize\_state\_source(init, cont\_params, model,

250  base\_rng, output,

251  context\_factory);

252  }

253  return false;

254  }

255  } // common

256 } // stan

257

258 #endif

stan::common::get\_double\_from\_string

bool get\_double\_from\_string(const std::string &s, double &val)

Converts string to double.

**Definition:** initialize\_state.hpp:208

stan::common::initialize\_state\_source

bool initialize\_state\_source(const std::string source, Eigen::VectorXd &cont\_params, Model &model, RNG &base\_rng, std::ostream \*output, ContextFactory &context\_factory)

Creates the initial state using the source parameter.

**Definition:** initialize\_state.hpp:154

boost::math::isfinite

bool isfinite(const stan::agrad::var &v)

Checks if the given number has finite value.

**Definition:** boost\_fpclassify.hpp:39

stan::common::initialize\_state\_random

bool initialize\_state\_random(const double R, Eigen::VectorXd &cont\_params, Model &model, RNG &base\_rng, std::ostream \*output)

Initializes state to random uniform values within range.

**Definition:** initialize\_state.hpp:82

write\_error\_msg.hpp

stan::model::gradient

void gradient(const M &model, const Eigen::Matrix< double, Eigen::Dynamic, 1 > &x, double &f, Eigen::Matrix< double, Eigen::Dynamic, 1 > &grad\_f, std::ostream \*msgs=0)

**Definition:** util.hpp:405

error\_codes.hpp

stan::common::write\_error\_msg

void write\_error\_msg(std::ostream \*error\_stream, const std::exception &e)

**Definition:** write\_error\_msg.hpp:11

stan::common::initialize\_state\_zero

bool initialize\_state\_zero(Eigen::VectorXd &cont\_params, Model &model, std::ostream \*output)

Sets initial state to zero.

**Definition:** initialize\_state.hpp:33

util.hpp

context\_factory.hpp

stan::common::initialize\_state

bool initialize\_state(const std::string init, Eigen::VectorXd &cont\_params, Model &model, RNG &base\_rng, std::ostream \*output, ContextFactory &context\_factory)

Creates the initial state.

**Definition:** initialize\_state.hpp:234

Eigen.hpp

stan::math::e

double e()

Return the base of the natural logarithm.

**Definition:** constants.hpp:86


---

[ Stan Home Page ]

*© 2011–2014,
Stan Development Team.*
